# Mindfulness training for adolescents: A neurodevelopmental perspective on investigating modifications in attention and emotion regulation using event-related brain potentials

**DOI:** 10.3758/s13415-015-0354-7

**Published:** 2015-04-07

**Authors:** Kevanne Louise Sanger, Dusana Dorjee

**Affiliations:** Centre for Mindfulness Research and Practice, Bangor University, Bangor, Gwynedd UK; School of Psychology, Bangor University, Adeilad Brigantia, Bangor, Gwynedd LL57 2AS UK

**Keywords:** Mindfulness, Attention, Development, ERP, Emotion regulation

## Abstract

Mindfulness training is increasingly being introduced in schools, yet studies examining its impact on the developing brain have been scarce. A neurodevelopmental perspective on mindfulness has been advocated as a powerful tool to enhance our understanding of underlying neurocognitive changes that have implications for developmental well-being research and the implementation of mindfulness in education. To stimulate more research in the developmental cognitive neuroscience of mindfulness, this article outlines possible indexes of mindfulness-based change in adolescence, with a focus on event-related brain potential (ERP) markers. We provide methodological recommendations for future studies and offer examples of research paradigms. We also discuss how mindfulness practice could impact on the development of prefrontal brain structures and enhance attention control and emotion regulation skills in adolescents, impacting in turn on their self-regulation and coping skills. We highlight advantages of the ERP methodology in neurodevelopmental research of mindfulness. It is proposed that research using established experimental tasks targeting ERP components such as the contingent negative variability, N200, error-related negativity and error positivity, P300, and late positive potential could elucidate developmentally salient shifts in the neural plasticity of the adolescent brain induced by mindfulness practice.

The term “mindfulness” was derived from the Buddhist concept of “sati,” which refers to refined skills of awareness, attention, and remembering (Siegel, Germer, & Olendzki, 2008). In the secular context, Bishop et al. (2004) proposed a two-component model of mindfulness, involving “self-regulation of attention” (maintained on immediate experience) and “attitudinal orientation” (curiosity, openness, and acceptance). They described mindfulness as a form of mental training that develops a reflective rather than reflexive mode of responding to internal and external events. Shapiro, Carlson, Astin, and Freedman (2006) later extended the model to include a third component: intention, which links back to the earlier notion of “remembering,” in addition to attention and attitude (IAA). They referred to Jon Kabat-Zinn’s description of mindfulness to explain their three axioms—“paying attention (Attention) in a particular way (Attitude): on purpose (Intention), in the present moment, and non-judgementally” (Kabat-Zinn, 1994, p. 4).

Several reviews have summarized the benefits of mindfulness for adult physical and mental health (Brown, Ryan, & Creswell, 2007; Eberth & Sedlmeier, 2012; Grossman, Niemann, Schmidt, & Walach, 2004), in various clinical populations (Baer, 2003; Hofmann, Sawyer, Witt, & Oh, 2010), and more recently in children, both within and outside a clinical context (Black, Milam & Sussman, 2009; Burke, 2010; Davidson et al., 2012; Greenberg & Harris, 2012; Meiklejohn et al., 2012; Zenner, Herrnleben-Kurz, & Walach, 2014). The field of mindfulness research is now moving toward more rigorous clinical trials, and an increasing number of studies are targeting the underlying neurocognitive mechanisms (Bradshaw, Goldweber, Fishbein, & Greenberg, 2012; Brown, Goodman, & Inzlicht, 2013; Chiesa, Calati, & Serretti, 2011; Hölzel et al., 2011; Lutz, Slagter, Dunne, & Davidson, 2008; Moore & Malinowski, 2009). Indeed, neuroscientific methods can help us understand the intricate relationship between cognition, neural plasticity, and mindfulness as a well-being-enhancing strategy. Better understanding, particularly from the neurodevelopmental perspective, may also inform the adaptation of interventions for specific populations such as adolescents, by taking into account their neurodevelopmental trajectories.

Davidson et al. (2012) outlined how contemplative practices including mindfulness could benefit young people at the interlinked levels of neural substrates, psychological functioning, and behavior. They discussed recent neurodevelopmental findings showing that social–emotional learning (SEL) programs implemented in regular school curricula improve executive function in children—for instance, in terms of inhibitory control and working memory (Greenberg & Rhoades, 2008)—and they emphasized that mindfulness-based approaches could further strengthen SEL-like programs. Specifically in adolescents, brain areas of executive function—that is, the prefrontal cortex (PFC), where emotion regulation networks overlap with circuitry for attention control (Ochsner & Gross, 2005; Lee, Heller, van Reekum, Nelson, & Davidson, 2012)—require greater activation than among adults for regulating emotions. Furthermore, high-arousal situations impair adolescent decision-making abilities much more than those in adults (Steinberg, 2005). These contingencies suggest that practices like mindfulness, which enhance executive functioning (Oberle, Schonert-Reichl, Lawlor, & Thomson, 2012; Westbrook et al., 2013), could support prefrontal brain structure development in adolescents. Arguably, such training could also enable adolescents to manage excessive levels of negative emotions like anxiety, which interfere with working memory and attention (Shackman et al., 2006; Shackman, Maxwell, McMenamin, Greischar, & Davidson, 2011) and impact on well-being and academic performance (Kaplan, Liu, & Kaplan, 2005). Mindfulness can also encourage a present-moment experiential form of self-awareness (ESA), as opposed to the traditional narrative form (NSA) (Davidson et al., 2012). Perceiving the self through ESA can decrease rumination and self-judgment, which in turn could benefit academic motivation and learning (Roeser & Peck, 2009). Davidson et al. (2012) exemplified this with beliefs about intelligence—perceiving intellect as a fixed trait was associated with higher test anxiety and poorer exam results, despite cognitive ability, whereas interventions that portrayed such constructs as being malleable could reduce anxiety and increase persistence and achievement (Dweck, 2008).

In summary, the neurocognitive perspective has great potential to help expand our understanding of the developmental mechanisms of mindfulness and their implications for the adaptation and implementation of specific adolescent programs, particularly in the context of education. The purpose of this article is to formulate the theoretical foundations for mindfulness research with adolescents, which would take into account both the complexities of training this age group and their neurodevelopmental dispositions. After discussing current mindfulness training programs for adolescents, we will outline some salient methodological issues, including the advantages of event-related brain potentials (ERPs) in developmental research on mindfulness. Then we will specifically focus on the potential of neurocognitive ERP markers of attention and emotion regulation in order to study the effect of mindfulness training in adolescence.

## Researching mindfulness-based programs for adolescents

Currently, several mindfulness-based programs for children and adolescents are available, which vary in their degrees of evidence-based support and their views about which aspects of mindfulness and related concepts (e.g., compassion, yoga, or cognitive behavior therapy) should be emphasized. These include Wellness Works in School (Kinder Associates LLC, 2002), Still Quiet Place (Saltzman & Goldin, 2008), Meditación Fluir (Justo, 2009), Mindfulness-Based Stress Reduction for Teens (MBSR-T: Biegel, 2009), Mindfulness for Adolescents (Dewulf, 2009), Mindful Schools (Mindful Schools, 2010), MindUp (The Hawn Foundation, 2011), Learning to BREATHE (L2B; Broderick, 2013), and the Mindfulness in Schools Project (MiSP; Kuyken et al., 2013). For a thorough discussion of the different programs and their outcomes, see Meiklejohn et al. (2012). One overarching feature of these programs is their Western cultural focus, which needs to be carefully considered if these programs are to be adapted for use in non-Western countries and cultures. This may impact on the format and underlying intentions of implementation, such as the enhancement of attention, well-being, and academic performance in the Western context, in contrast to the encouragement of cognitive flexibility and creativity in some Asian cultures. Given the nascent nature of these programs, many of them lack strong research evidence, most having only one peer-reviewed study to support their efficacy. One of the methodologically strongest studies conducted so far was by Biegel, Brown, Shapiro, and Schubert (2009), who evaluated the MBSR-T intervention with young people receiving community mental health support. The study followed a randomized controlled design, including a posttreatment follow-up and diagnostic assessments by a study-blind clinician. The participants in the training group significantly improved on levels of anxiety, perceived stress, and depression by the 3-month follow-up, and also showed diagnostic improvements. Hopefully, more future studies will apply as robust a design as this trial, while also integrating neurocognitive assessments that have not so far been employed.

It is also important to consider the contribution of participant-specific variables to the outcomes of studies with adolescents. For example, the significant findings in Biegel et al. (2009) could have been impacted by the clinical nature of the sample—a variable that needs to be taken into account in future research with nonclinical populations in schools where the ceiling effect may decrease the likelihood of finding improvements. Indeed, Gould, Dariotis, Mendelson, and Greenberg (2012) reported just this, finding that baseline depression scores moderated mindfulness-training effects in urban youths. This highlights the need for measures that would be sensitive enough to detect changes in both clinical and nonclinical groups—which is where neurocognitive assessments can be particularly helpful. In addition to the clinical–nonclinical differences, personality traits have been found to impact on the outcomes of mindfulness training with adolescents. Specifically, Huppert and Johnson (2010) reported that agreeableness and openness significantly correlated with improvements in well-being after MiSP training. In addition, nonreactivity has been reported to directly moderate the beneficial effects of mindful observing on anxiety and depression (Desrosiers, Vine, Curtiss, & Klemanski, 2014). Age may also play a role; Schonert-Reichl and Lawlor (2010) found in their early validation study of MindUp (previously known as Mindfulness Education) that the course was more effective with younger students.

Two other issues for consideration are the amount of home practice necessary for a significant effect and the required qualifications of a program’s trainers. One of the most prevalent adult-based mindfulness training programs is Mindfulness-Based Stress Reduction (MBSR; Kabat-Zinn, 1990), which involves eight 2.5-h sessions and asks participants to individually practice for 45 min daily. These requirements are reduced in most youth mindfulness programs, but there is still a strong emphasis on regular practice, if only for 5–10 min per day. It is currently not clear how the amount of home practice impacts on outcomes with this population. Foret et al. (2012) trained 11th-grade students (16–17 years old) in a 4-week relaxation curriculum including mindfulness and breathing meditation. After the intervention, the pupils reported significant improvements in their stress management skills, perceived stress, and state anxiety. These promising results were found in a group in which 80% either never logged on to the website for their home practice or only completed it once. However, Huppert and Johnson (2010) found that home practice was a significant contributing factor to mindfulness and well-being effects after MiSP training. The latter intervention was run with a younger (14–15 years), all-male cohort. It is possible that younger adolescents who are less matured neurologically and behaviorally, or boys, who are known to have a slower developmental trajectory (Brumback, Arbel, Donchin, & Goldman, 2012), need this additional practice. Methodologically, home practice is a pivotal issue, given that it may have a significant impact on intervention effects (Zenner et al., 2014), but is difficult to record accurately. Researchers have typically relied on self-report measures, but the use of ID-locked online home practices (e.g., Foret et al., 2012) or a similar tracking system is a potential solution. More reliable measures would enable us to fully review the extent of home-practice contributions and allow program designers to balance intervention efficacy with time effectiveness.

In terms of trainer qualification requirements, few studies rigorously report on the mindfulness training and qualifications of the program teachers. Most mindfulness-based programs for adolescents involve external trainers, normally a program’s developers, communicating the mindfulness course to pupils (e.g., Broderick & Metz, 2009; Huppert & Johnson, 2010; Kuyken et al., 2013; Raes, Griffith, Van der Gucht, & Williams, 2014). But if mindfulness programs prove effective enough to be considered for broad implementation into school curricula, mindfulness training and delivery will need to become more self-sustaining. Most education boards cannot afford to pay external trainers in the long term, but without upholding the fidelity of the program, any attempts at training young people may be fruitless. An alternative model would involve training school teachers in mindfulness, supporting them to develop a personal practice, and then training them in the delivery of mindfulness-based curricula (e.g., the model developed and used by the Centre for Mindfulness Research, & Practice, 2014). It seems that a compromise between these two delivery approaches could be met, in order to achieve a program that all schools could practically accommodate without weakening the therapeutic outcomes.

## Methodological issues of neurodevelopmental research on mindfulness with adolescents

Bradshaw et al. (2012) evaluated the theoretical aspects of developmental neuroscience that could help shape school-based preventative interventions—developmental research on reward pathways, stress, social–cognitive mechanisms, and executive functioning seem particularly relevant here. They concluded that mindfulness-based programs could help promote regulatory processes if they were taught within the education system. However, preliminary studies with children, including two systematic reviews, only reported low to moderate effects (Black et al., 2009; Burke, 2010; Flook et al., 2010). In comparison, meta-analysis reports in adults, evaluating 8-week courses in MBSR (Kabat-Zinn, 1990) or mindfulness-based cognitive therapy (MBCT; Segal, Williams, & Teasdale, 2002), have found medium to high effect sizes on various measures of mindfulness, well-being, negative affect, and so forth (Grossman et al., 2004; Hofmann, Sawyer, Witt, & Oh, 2010). This could, in part, result from the developmentally appropriate reductions to course length and depth, as well as in practice frequency and duration, seen in child mindfulness programs. In this context, the adolescent age group may be a more advantageous research population than younger children, bridging the gap between adult and child mindfulness-based curricula. Adolescence can also be seen as a late catchment point for neurocognitive interventions within the context of prefrontal development (Spear, 2013), with mindfulness training particularly targeting attention-monitoring and control systems of the PFC (Hölzel et al., 2011).

Spear (2013) also advocated for adolescent-related governmental policy to take more consideration of developmental neuroscience. This position was based on the emerging consensus among researchers that adolescents’ perceptions of rewarding and aversive stimuli are strongly biased toward the former, and that the role of context is highly influential over their decision-making abilities. Spear reviewed recent fMRI research and nonhuman animal models that demonstrated that adolescents are susceptible to “hot cognitions,” in which the still-maturing prefrontal cortex can process cognitive tasks at adult levels of performance but can also be overridden by more-matured subcortical brain regions of the dopaminergic, mesolimbic reward pathway, like the ventral striatum. This shift in activation can result in greater risk-taking behaviors (Gardner & Steinberg, 2005). Spear ends by proposing that developmental neuroscience could help introduce interventions that actively support the development of prefrontal brain regions, thus balancing adolescent perceptions of reward and punishment in order to prevent extreme risk-taking. Mindfulness-based training could be a helpful intervention in this context, particularly if it can be sustainably implemented in schools.

The introduction of developmental neurocognitive approaches into mindfulness training and research requires careful consideration of suitable research tools. Investigations of neurocognitive mechanisms in adults have so far mostly relied on functional and structural magnetic resonance imaging (MRI; see Hölzel et al., 2011), and to a lesser extent on ERPs, which are averaged brain waves in response to certain categories of stimuli, such as oddballs or affective pictures (Fabiani, Gratton, & Coles, 2000). Each of the two methods has its pros and cons, with MRI having superior spatial resolution, and thus being more suitable for research questions about localization, and ERPs providing millisecond temporal accuracy of neurocognitive processing, which is advantageous for questions tapping into automatic versus controlled processing. Another strength of MRI is its longer-standing use in mindfulness research, so that the grounding for further developments is already established. Although less frequently used in mindfulness research so far, the ERP methodology has the advantage of being less costly and theoretically more firmly established, after more than 60 years of research. Importantly, ERP research has identified functionally specific indexes of neurocognitive processing, so called *ERP components*, which can be used in the assessment of attention control and efficiency, inhibition, semantic processing, and error judgment. In addition, ERPs can be particularly helpful in the investigation of skill acquisition effects, where fMRI findings focusing on localization may mask functionally salient changes in processing efficiency induced by learning (Dorjee & Bowers, 2012). Nevertheless, developmental factors must be taken into account if one is using either approach. ERP components have typically been described in adult populations, whereas the limited child research has indicated different scalp localizations and time courses in the still-developing brain. Please refer to Table 1 for studies in which these differences are also known to apply in adolescence. The following ERP components seem particularly relevant to neurocognitive research on mindfulness with adolescents:

**Table 1:** ERP components of interest and corresponding research summary table

Event-Related Potential	Definition	Experimental Paradigm / Task	Adolescent Patterns	Adult Patterns	Predictions for Adolescents Trained in Mindfulness Practices
Contingent negative variation (CNV)	A slow-building negative waveform that emerges in the period between a warning and imperative stimuli. It has a frontal/central distribution, and signals preparatory motor activity, sensory anticipation, and the activation of attention (Walter et al., 1964).	• Priming task with informative and noninformative warning stimuli (primes)	• Killikelly and Szucs (2013) proactive response activation toward an imminent target, which correlates with more negative CNV amplitudes, is dampened in older adolescents compared to young adults.	• Bostanov et al. (2012)—mindfulness practice leads to a more negative CNV in depressed adult populations, who have dampened preintervention CNV activity before the intervention.	• The CNV can detect facilitation of anticipatory attention processes after mindfulness interventions, as indicated by converging evidence on developmental CNV modulation in adolescents (Killikelly & Szucs, 2013) and its modifiability through mindfulness training in depressed populations (Bostanov et al., 2012). • More negative CNV amplitudes could be expected after training.
Visual N2	The visual N2 is a negative deflection with frontal/central distribution, and it signals task-specific inhibition (Falkenstein et al., 1999) and conflict monitoring (Ladouceur et al., 2007). The N2 can also be detected at occipital sites (Fabiani et al., 2000).	• Inhibition or detection task • Stroop test (Stroop, 1935) • Go, no-go task (Donders, 1969) • D2 (Brickenkamp, 1962) • Attention Network Test, conflict-monitoring task (Fan et al., 2002) • Odd-ball paradigms	• Chapman et al. (2010) mapped out N2 development, detecting more negative amplitudes and shorter latencies in adolescents compared to children. This suggests more efficient and mature processing in older adolescents, comparable to adult levels.	• Moore et al. (2012)—more negative N2 response after mindfulness training in Stroop task. This modulation has also been linked to reduced P3 positivity, suggesting that stronger inhibition reduces the cost of attentional resources on goal-irrelevant stimuli.	• More negative N2 amplitudes could be expected after mindfulness training in adolescent populations, reflecting more mature patterns of inhibition (Chapman et al., 2010). • N2 amplitudes should correlate with P3 responses and performance accuracy in odd-ball or SART (Robertson et al., 1997) paradigms, given that increased N2 may result in a reduction of P3b amplitude while maintaining or increasing task accuracy and vigilance. • Possible links between N2 modulation and well-being since better inhibition has partially mediated increased well-being in adults (Sauer, Walach, & Kohls, 2011).
P300 *Specific interest in P3b sub-component	The P300 (P3 family of positive components) is elicited in response to salient stimulus events, thus allocating mental resources to attention and memory operations associated with stimulus processing (Polich, 2007). The P3b specifically has a centro-parietal distribution and is linked to stimulus categorization and processing, as well as updating working memory (Sutton et al., 1965).	• Focused attention and executive tasks • Dual-task paradigms e.g. *n*-back (Watter et al., 2001) and task switching (Kieffaber & Hetrick, 2005) • Attentional-blink paradigm (Raymond et al., 1992) • Stroop (Stroop, 1935) • Odd-ball paradigm • SART (Robertson et al., 1997)	• Brumback et al. (2012) the developmental maturity of P300 amplitude and latency responses during a visual odd-ball task develops sooner in females (i.e. shorter P300 latency and less positive P300 waveforms). This suggests that males have a slower developmental trajectory, and executive attention in particular is still developing well into older adolescence.	• Slagter et al. (2007)—improved T2 detection after mindfulness training in the attentional-blink task. Performance was associated with reduced T1-elicited P3b amplitude over time, suggesting more efficient orientating and alerting. • Moore et al. (2012)—P3b amplitude reduction during Stroop task on incongruous targets after mindfulness training. This suggests a reduced cost on attention resources for target inhibition. • Smallwood et al. (2008) less mind wandering correlates with greater P3b mean amplitudes during the SART, suggesting more consistent attention processing. Mind wandering has also been shown to reduce after mindfulness practice (Mrazek et al., 2012) suggesting that P3b amplitudes would be a useful psycho-physiological marker of mind wandering reduction after mindfulness training.	• Attentional-blink paradigm is a possible ERP marker to map the development of orientating and alerting performance in adolescents, and could be particularly useful in relation to high impulsivity (Ray Li et al., 2005), reflected in improvements to T2 detection and associated decreases in P3b to T1. • Palluel et al. (2010) 14–15 years is the turning point for dual-task performance. Thus, adult levels of performance could be anticipated in older adolescents, reflected in reduced P300 amplitudes in Stroop or task-switching paradigms. • Where postintervention P3b amplitude is reduced, a corresponding increase is anticipated for early components—e.g., CNV or N2—signaling better preparation, detection, or inhibition. • Similar mind wandering effects could be anticipated in adolescents as in adults (Mrazek et al., 2012; Smallwood et al., 2008), impacting learning and academic performance.
Error-related negativity (ERN) and error positivity (Pe)	ERN is a negative deflection over fronto-central sites, signifying attention to an error deviating from goal-directed behavior (Falkenstein et al., 1991). This is followed by error Positivity (Pe), which is sensitive to the subsequent appraisal of error severity (Overbeek et al., 2005).	• Any task requiring accuracy, with higher likelihood of errors that can be detected or alerted to • Stroop task (Stroop, 1935) • Flanker task (Eriksen & Eriksen, 1974)	• Ladouceur et al. (2007) reported that Pe amplitudes showed a developmental peak in older adolescents, responding to errors with more positive amplitudes than either young adolescent or adult participants.	• Long-term effects of meditation on ERN and Pe have been tested by Teper and Inzlicht (2013). ERN negativity positively correlated with meditation practice, and emotional acceptance, without an amplitude increase in Pe, following Stroop task errors. • Larson et al. (2013), a cross-sectional study of nonmeditators following either a guided mindfulness CD or educational talk. Mindfulness induction corresponded with reduced Pe mean amplitudes after errors on the Eriksen flanker task.	• Accurately identifying errors (more negative ERN) without the upsurge in emotional response (no change or less positive Pe amplitude), might allow adolescents to learn from their mistakes without self-punishment. This mechanism could have links to reducing hypercritical self-judgment that can otherwise result in extreme risk-taking and self-harm (Klonsky, 2007).
Late positive potential (LPP)	A slow-building positive waveform, with a central/posterior distribution. Considered to be a neural marker of emotional arousal, reflecting preferential processing and encoding of motivationally relevant stimuli (Brown et al., 2013).	• Passive viewing or hearing of affective stimuli • Affect labeling task • Affect induction / regulation tasks (e.g. Hajcak & Nieuwenhuis, 2006)	• LPP response modulation does not reach adult levels until late adolescence (Zhang et al., 2012). Developmental increases indicate more adaptive attention processing. • Zhang et al. (2013), peak emotional ratings of pictures and LPP amplitudes reported in young adolescents, corresponding to the undeveloped nature of their ER abilities.	• Brown et al. (2013) found a strong inverse correlation between dispositional mindfulness and the LPP amplitude elicited by high-arousal, unpleasant stimuli in a passive International Affective Picture System (IAPS) picture-viewing task.	• Mindfulness training may impact on the strength of emotional responses, reflected in LPP amplitude reduction (possibly linked to less arousal and better emotion regulation), in the young adolescent population (Zhang et al., 2013).
Late positive complex (LPC)	Positive, broad-peaking waveform with a parietal distribution. It is associated with deep processing (Rellecke et al., 2012), memory recall (Schaefer et al., 2011), and affective rumination (Sitges et al., 2007).	• Passive viewing or hearing affective stimuli • Negative emotion “priming” tasks, combining CNV and LPC neural markers to map regulation through initial attention deployment (CNV), followed by reduction in rumination (LPC) • Affect labeling task	• Marks et al. (2010) found that higher levels of rumination exacerbate the relationship between self-reported life hassles and depression and anxiety, whereas higher dispositional mindfulness can alleviate these same links.	• Rellecke et al.’s (2012) LPC mean amplitude increases were induced only when participants intentionally focused attention on affective facial stimuli, indicating individual’s voluntary control over higher cognitive emotion processing.	• Initial correlational studies measuring dispositional mindfulness, trait rumination and the LPC in adolescents could provide useful insights; increased rumination and decreased mindfulness should be linked to less positive LPC amplitudes. • Training adolescents in mindfulness techniques may protect them against the damaging effects of rumination on well-being (Marks et al., 2010), reflected in reduced LPC. • Future studies could test the process of change in ER using LPC and CNV ERP neural markers.

1. The contingent negative variation (CNV) is a slow-building negative waveform that emerges in the period between a warning and imperative stimulus. It has a frontal/central distribution and signals preparatory motor activity, sensory anticipation, and the activation of attention (Walter, Cooper, Aldridge, McCallum, & Winter, 1964).
2. The visual N200 (N2) is a negative deflection with a frontal/central distribution, which signals task-specific inhibition (Falkenstein, Hoormann, & Hohnsbein, 1999) and conflict monitoring (Ladouceur, Dahl, & Carter, 2007). It can also be detected at occipital sites (Fabiani et al., 2000).
3. The P300 (P3 family of positive components) is elicited in response to salient stimulus events, thus allocating mental resources to attention and memory operations associated with stimulus processing (Polich, 2007).
4. Two subcomponents of the P300, the P3a and P3b, have been distinguished. Whereas the P3a has a fronto-central distribution and is associated with automatic attention capture (Polich, 2007), the P3b has a centro-parietal distribution and has been linked to stimulus categorization and processing, as well as to an updating of working memory (Sutton, Braren, Zubin, & John, 1965).
5. The error-related negativity (ERN) is a negative deflection over fronto-central sites, used to assess automatic responses to an error that deviates from goal-directed behavior (Falkenstein, Hohnsbein, Hoormann, & Blanke, 1991). This is followed by the error positivity (Pe), which is sensitive to the subsequent appraisal of that an error’s severity (Overbeek, Nieuwenhuis, & Ridderinkhof, 2005).
6. The late positive potential (LPP) is considered a neural marker of emotional arousal, reflecting the preferential processing and encoding of motivationally relevant stimuli. It is a slow-building positive waveform with a central/posterior distribution (Brown et al., 2013).
7. The late positive complex (LPC) is a positive, broad-peaking waveform with a parietal distribution. It has been associated with deep processing (Rellecke, Sommer, & Schacht, 2012), memory recall (Schaefer, Pottage, & Rickart, 2011) and affective rumination (Sitges et al., 2007).

We will discuss each of these components in relation to the effects of mindfulness on attention and emotion processing in adolescents in the following sections.

## Mindfulness training effects on attention

Attention is a key modulator of cognitive processing, enabling us to select task-relevant stimuli and inhibit irrelevant information, sustain focus on cognitive performance, divide attention, and stay vigilant when needed. One theory of attention that is commonly used in mindfulness research proposes that there are three largely independent attention networks (Petersen & Posner, 2012; Posner & Petersen, 1990). The *orienting network* supports selecting relevant sensory input and shifting attention toward a goal-related stimulus (disengaging and reengaging attention). The *alerting network* refers to a level of arousal and vigilance—that is, the ability to maintain sensitivity to external stimuli. This can be further separated into *phasic alertness*, which is task-specific, and *intrinsic alertness*, which refers to more general trait levels of arousal. Finally, *executive attention* monitors and resolves any conflicts arising between thought, emotion, and action (Raz & Buhle, 2006). Executive attention is typically measured by using tasks involving an incompatibility between different aspects of a task, otherwise known as “conflict,” such as in the classic Stroop task, in which an individual must ignore the written word (e.g., RED) and instead identify the color that the word is written in (e.g., blue; Stroop, 1935). Conflict monitoring is believed to be one key aspect of executive attention, along with computing task parameters, evaluating conflict, updating new or unlearned responses, regulating thoughts and emotions, and blocking habitual responding (Raz & Buhle, 2006). Although a body of research supports both functional and neural separation of these networks, there is also evidence that it can be difficult to methodologically separate the attention networks, because they often operate in parallel (MacLeod et al., 2010). The majority of studies regarding attention performance and mindfulness practice have discussed improvements in terms of the three attention networks (Chan & Woollacott, 2007; Chiesa, Calati, & Serretti, 2011; Jha, Krompinger, & Baime, 2007; Malinowski, 2013; Tang, Hölzel, & Posner, 2015), and in order to evaluate the past literature consistently, we will also apply this approach. However, there are other frameworks within the broader attention literature, with the integrated competition hypothesis being another common explanation supported by neurocognitive findings (Desimone & Duncan, 1995; Duncan, Humphreys, & Ward, 1997). For a more in-depth discussion of the ERP literature on attention, please refer to Luck (2014) or Picton et al. (2000).

In addition to task-specific attention improvements, we will give special consideration to past research and ERP markers of mind wandering. This is defined as the withdrawal of attention from task-relevant stimuli, which subsequently reduces the capacity of working memory. Mind wandering involves brain regions responsible for orienting, goal-directed behavior, and executive attention (Smallwood, Beach, Schooler, & Handy, 2008). It has previously been linked to reductions in learning and task performance, as well as to clinical issues such as depression (Deng, Li, & Tang, 2014; Mooneyham & Schooler, 2013), and is therefore a process of particular interest to schools, given that mind wandering is known to occur most often in educational contexts (Unsworth, McMillan, Brewer, & Spillers, 2012).

## ERP markers of attention and experimental paradigms for mindfulness research with adolescents

### The CNV

The contingent negative variation represents attention activation, motor preparation, and a proactive response to suggested targets. It has a frontal/central distribution and has been linked to activation in the anterior cingulate cortex (ACC), PFC, and supplementary motor area (SMA) (Bares, Nestrasil, & Rektor, 2007; Nagai et al., 2004). These brain regions have also been associated with increased activation during meditation in trained practitioners (Hölzel et al., 2007), which may signal that mindfulness practice benefits mental alertness (Posner, 2008). Tasks such as the Attention Network Test (ANT: Fan, McCandliss, Sommer, Raz, & Posner, 2002) identify this orienting attention ability through measuring the response when spatial cues appear before an impending target. Jha, Krompinger, and Baime (2007) demonstrated that the orienting of attention is significantly improved in adults after an 8-week MBSR course.

The CNV might be a sensitive ERP component to detect possible facilitation of attention “priming”; for instance, Bostanov, Keune, Kotchoubey, and Hautzinger (2012) demonstrated that mindfulness practice can increase CNV negativity in depressed adult populations. Interestingly, Killikelly and Szucs (2013) showed that proactive response activation toward an imminent target, which correlated with more-negative CNV amplitudes, is reduced even in older adolescents, relative to young adults. Adolescents’ suggested lack of orientation and proactive planning abilities, associated with a less-negative CNV (and less-efficient frontal lobe executive functioning), has potential consequences for risk-taking behaviors. This also means that testing paradigms aimed at manipulating the CNV will likely show poorer baseline functioning in adolescent participants than in adults. Nevertheless, this converging evidence on developmental CNV modulation in adolescents (Killikelly & Szucs, 2013)—as well as the CNV’s modifiability through mindfulness training in depressed populations (Bostanov et al., 2012), who also show dampened pretesting CNV responses—recommends this as a sensitive tool for understanding the underlying mechanisms of mindfulness-based change. More-negative CNV amplitudes could be expected after mindfulness training in adolescents, reflecting anticipatory mobilization of attentional resources.

### The N200

Response inhibition is a strong contributor to performance in tasks of executive attention such as the Stroop (1935), go–no-go (Donders, 1969), and D2 (Brickenkamp, 1962) tasks, as well as the conflict subtask of the ANT, and can be monitored through the N200 (N2) negativity. Moore, Gruber, Derose, and Malinowski (2012) employed the Stroop task with mindfulness novices before and after an 8-week program (and at a 16-week follow-up), which involved an initial 2-h guided session and regular 10- to 15-min practices 5 days per week. No behavioral effects were found, but N2 amplitudes showed a significant Group × Time interaction (at occipito-parietal electrode clusters), which reflected more-negative amplitudes in the meditation group by follow-up. There was also a significant decrease in meditators’ P3b amplitudes during incongruent trials. The authors suggested that the N2 amplitude changes indicated improvements in detection and inhibition, which subsequently led to fewer attentional resources being required for object recognition, reflected in less-positive P3b amplitudes. In other words, efficiently allocating cognitive resources to early detection left mental resources for maintaining task vigilance. In this context, the N2 can thus be interpreted as a direct index of response inhibition and can indicate the facilitation of improved orienting (attention shift) and alerting (vigilance) skills after mindfulness training. The nonsignificant group effect for performance highlights the idea that changes in the alerting system may be subtle, needing prolonged practice before enhanced attention leads to significantly more-accurate task performance. This favors neurocognitive methods of testing such as ERPs, which may identify early mechanisms of change. Ortner, Kilner, and Zelazo (2007) also reported that university students practicing mindfulness were better able to disengage from unpleasant stimuli, in comparison to those in a passive control or relaxation group. However, Ortner et al. observed no difference between groups when observing neutral or pleasant stimuli, suggesting that mindfulness practice does not uniformly disengage people from experience, but rather, prevents interference by negative stimuli. Future testing methods may correlate N2 and P3b amplitudes, as well as performance accuracy, using an odd-ball or “sustained attention to response” task (SART; Robertson, Manly, Andrade, Baddeley, & Yiend, 1997).

With regard to adolescents, studies have shown that they have difficulty shifting attention away from emotive stimuli (e.g., Shackman et al., 2011). Being taught how to effectively reengage their attention through mindfulness could improve concentration levels at school, where social, emotional, and academic stressors are widespread and distracting. Bogels, Hoogstad, van Dun, de Schutter, and Restifo (2008) found that after an MBCT course, adolescents with externalizing disorders showed significant improvements on speed and accuracy measures of sustained attention (D2 test of attention; Brickenkamp, 1962), with further improvements by an 8-week follow-up. Caution is needed when generalizing findings from clinical samples, but these results suggest that mindfulness can enhance sustained attention in this age group. In terms of typical development, Chapman, Woltering, Lamm, and Lewis (2010) mapped out N2 development in participants ranging from 8–17 years old. They detected more-negative amplitudes and shorter latencies in adolescents, which suggests more-efficient and -mature processing, akin to adult levels. Therefore, similar to what has been reported in adult populations, more-negative N2 amplitudes could be expected after mindfulness training in clinical and nonclinical adolescent populations, reflecting enhanced patterns of automatic attention capture and inhibition.

### The P300

The attentional-blink paradigm (Raymond, Shapiro, & Arnell, 1992) is another measure of orienting and alerting performance. This measures an individual’s ability to disengage from Target 1 (T1) and stay vigilant for detecting Target 2 (T2), both presented quickly (less than 500 ms apart) in a visual sequence of numbers and letters. In relation to mindfulness, Slagter et al. (2007) found significantly better T2 detection in participants after a 3-month mindfulness meditation retreat, as well as a selective reduction in T1-elicited P3b amplitudes on no-blink (i.e., successful T2 detection) trials. These results suggest that reducing attentional resources to T1 through meditation training increased an individual’s chances of remaining vigilant and detecting T2. In terms of relating this to adolescents, Brumback et al. (2012) demonstrated that the maturity of the P300 amplitude and latency develops earlier in females—that is, reduced P300 positivities and shorter latencies. This suggests that male students may have more potential to improve attention performance after training, but also that baseline differences in mixed-gender groups should be controlled for. Research with adolescents specifically using the attentional-blink paradigm (Ray Li, Chen, Lin, & Yang, 2005) has reported that impulsivity was associated with lower T2 detection. Improvements in adults (Slagter et al., 2007) would therefore suggest that mindfulness training might be particularly beneficial for adolescents with high impulsivity, with practice benefits being reflected in greater T2 detection and associated P3b amplitude decreases to T1.

The P3b reduction found after mindfulness training by Moore et al. (2012) during inhibition trials in the Stroop task reflects conflict monitoring and inhibitory performance in addition to alerting, discussed in the previous section. MBCT has also been shown to enhance executive control abilities in healthy adults (Chambers, Lo, & Allen, 2008). Moore et al. (2012) showed no corresponding drop in task accuracy when P3b amplitudes decreased, suggesting a reduction in the amount of attentional resources required for executive control. However, Moore and Malinowski (2009) reported in a cross-sectional study, with meditation-naive participants being compared to intermediate Buddhist students (who had at least completed the 6-week intensive course), that meditation students did perform significantly better in Stroop and D2 tasks. These improvements indicate better inhibitory control, cognitive flexibility, and quality of performance that might reflect the more intense nature of their meditation training. Other executive function tests use dual-task paradigms, such as the *n*-back paradigm (Watter, Geffen, & Geffen, 2001) and task switching (Kieffaber & Hetrick, 2005), which also modulate the P300. In dual-task paradigms, increases in task difficulty (including task switching) result in more-positive P300 amplitudes and prolonged latencies (Kramer, Wickens, & Donchin, 1985). Palluel, Nougier, and Olivier (2010) identified 14–15 years of age as the maturational turning point in dual-task performance, so that adults levels of performance should be anticipated in older adolescents unless affective stimuli are incorporated that prompt subcortical brain regions to interfere with executive functioning (Spear, 2013).

In contrast to the increased efficiency of attention resulting from mindfulness training, mind wandering is marked by a lack of efficient attention allocation. This can have a detrimental impact on education, in terms of both effective learning processes and academic performance. For example, during revision periods, an awareness of an individual’s optimum attention span and an ability to catch themselves mind wandering could maximize both productivity and a sense of achievement. A person’s susceptibility to mind wander can be measured using such simple, overlearned tasks as the SART (Robertson et al., 1997), in which behavioral performance correlates with self-reported mind wandering and P3b waveforms. Improvements in dual-task performance (which is essentially the internal environment that mind wandering creates) have been associated with activity in the ACC (Dreher & Grafman, 2003), one of the key brain areas linked to mind wandering (Ros et al., 2013) and mindfulness practice (Hölzel et al., 2007). Less mind wandering correlates with greater P3b mean amplitudes to nontargets preceding a correctly inhibited response to target during the SART, suggesting more-consistent attention processing throughout (Smallwood et al., 2008). This contrasts with the reduced P3b that is observed after mindfulness training in other attention-based tasks, thus highlighting the importance of context in neurocognitive theory and testing. Mind wandering has been shown to be reduced after brief mindfulness practice in adults (Mrazek, Smallwood, & Schooler, 2012), and similar effects could be expected in adolescents, positively impacting on the effectiveness of their learning. Moreover, it would be very relevant to investigate whether temporary improvements such as those reported by Mrazek et al. could, with regular mindfulness training, translate into stable, trait-like reductions in mind wandering.

## Mindfulness training effects on emotion regulation

Emotions are multifaceted phenomena involving the cognitive appraisal of a situation, physiological responses, emotional experience (feelings), and a tendency to respond (Scherer, 2000). The ability to manage emotions is described as *emotion regulation* (ER; Gross & Thompson, 2007). Typically, emotions compete for attention with other cognitive processes that are demanded by a situation, and the prefrontal systems of attention control enable the inhibition, shifting of attention, and monitoring of emotions that underlie ER (Ochsner, Bunge, Gross, & Gabrieli, 2002). This malleability by attention processes makes ER subject to maturation changes throughout development.

The links between attention control and ER have consequences for the adolescent population, since adolescents typically display poorer inhibition and more impulsive, risk-taking behaviors. Specifically, Ernst, Pine, and Hardin’s (2006) triadic model of motivated behavior illustrates how the adolescent reward-driven system (ventral striatum) may exert more control over adolescents’ behavior than the harm-avoidant (amygdala) and regulatory control (medial/ventral PFC) system that is still developing. In addition, reward regions of the brain are more strongly activated when young people take risks in the company of their peers (Steinberg, 2010), confirming the strong impact of peer pressure on this population. Gambling tasks such as that of Van Leijenhorst et al. (2010) exemplify that the adolescent ACC, which balances emotions with objective judgment, exerts less control over behavior than does the reward-oriented ventral striatum. This leads to riskier decisions based on the different developmental trajectories of these neural systems.

The connection between attention control and ER through the ACC is paramount to where mindfulness can influence ER strategies (Dywan, Mathewson, Choma, Rosenfeld, & Segalowitz, 2008). It is possible that mindfulness practice could encourage connections between the relevant prefrontal structures in adolescents, stabilizing arousal and reducing harmful risk-taking, as has been advocated more broadly in support of mental training by Atkins, Bunting, Bolger, and Dougherty (2012). Relevant evidence for this has come from adult fMRI research; for example, Grant, Courtemanche, and Rainville (2011) found that proficient meditators display less activation in brain regions of executive functioning (lateral and medial PFC, ACC) while attending to pain. This may represent a selective reduction in unhelpful cognitive elaboration, translating into more effortless ER. Petter, McGrath, Chambers, and Dick (2014) reported similar behavioral findings, with mindfulness practice attenuating pain responses in typical adolescents. The influence of mindfulness practice on ER strategy is complex, however, and fMRI research has shown a shift in ER strategies with more extensive mindfulness experience. Taylor et al. (2011) reported that actively engaging in mindful meditation during negative picture displays caused bilateral deactivation of the amygdala for beginners, whereas long-term practitioners showed a deactivation in the left lateral PFC. Meditators may initially reappraise in order to modify maladaptive emotional responses, but progressively an all-accepting attitude leaves appraisal unnecessary (Teper, Segal, & Inzlicht, 2013). Mapping the development of ER in adolescents will therefore need to take into account neurocognitive changes in relation to the length and quality of practice. The task designs and stimuli used will also influence the predictions made. During studies of pain tolerance, it is most adaptive to disengage from the incoming stimulus, and therefore less activation of the PFC may be recorded. Whereas, during an active ER task—for example, that of Creswell, Way, Eisenberger, and Lieberman (2007)—individuals must first appraise stimuli for valence and then actively down-regulate the emotional response that this has upon the amygdala.

In the next section, we will review previous ERP research, outline specific testing paradigms, and suggest what neural markers sensitive to ER could be studied within the adolescent population.

## ERP markers of emotion processing and experimental paradigms for mindfulness research with adolescents

### The P300

In comparison to MRI research, the utilization of ERPs to interpret emotion regulation is still emerging. However, the applications of the P300—described by Polich (2007) as having associations to executive attention, memory updating and retrieval (the P3b in particular), the dopaminergic reward system, and the PFC and ACC—all point to its relevance for ER. In particular, the P3b component has been linked to cognitive processing during emotion discrimination tasks, showing sensitivity to modulation by stimulus valence and intensity (Cavanagh & Geisler, 2006), as well as arousal (Briggs & Martin, 2009). The P300 has been shown to detect mindfulness effects in cognitive attention tasks such as the attentional-blink and the Stroop task with neutral stimuli (e.g., Moore et al., 2012; Slagter et al., 2007), indicating that the P300 may also prove a sensitive marker for changes to affective orientation linked to the efficiency of attention processing.

### The ERN/Pe

ER strategies associated with the ACC can be examined through the ERN ERP component, which is followed by the Pe—both elicited in trials in which participants make and review an error (van Veen & Carter, 2002). There has been some discussion over the specific role of the ERN, since Endrass, Reuter, and Kathmann (2007) found no difference in ERN responses whether or not the error was noticed by the individual, whereas the Pe indicated more conscious error awareness. The ERN may be considered a neural correlate of conflict monitoring, signaling the need to increase cognitive control and modulate behavior (Botvinick, Braver, Barch, Carter, & Cohen, 2001). A more negative ERN has been associated with general anxiety (Hajcak, McDonald, & Simons, 2003) and negative mood (Hajcak, McDonald, & Simons, 2004). The Pe component was unaffected during the anxiety study, and less positive Pe waveforms were reported in Hajcak et al. (2004) for a high-negative-affect group. The latter result was thought to indicate less salience for the individual when making an error. Interestingly, a cross-sectional study of nonmeditators following either a guided mindfulness practice CD or an educational talk demonstrated that the mindfulness induction also had the effect of reducing Pe mean amplitudes during error trials in a flanker task (Larson, Steffen, & Primosch, 2013). This finding is particularly significant for older adolescents, who typically respond to errors with more-positive Pe amplitudes, signaling stronger affective responses than among either adults or children (Ladouceur et al., 2007). It could be expected that the kind, accepting attitude of mindfulness contributed to a less positive Pe coupled with an adequate response to error (ERN), in contrast to the apathetic Pe reduction seen in people with high negative affect. This is where sensitive self-report measures of error judgments in conjunction with ERP testing might be able to identify the mediating cause behind Pe modulation after mindfulness practice. The long-term effects of meditation on the ERN and Pe have been tested by Teper and Inzlicht (2013), with ERN negativity being positively correlated with meditation practice, without an amplitude increase in the Pe. Accurately responding to errors (ERN) without the upsurge in emotional response (Pe) might allow adolescents to learn from their mistakes without self-punishment, a mechanism with clear links to reductions in risk-taking and self-harm that can otherwise result from hypercritical self-beliefs and negative affect (Klonsky, 2007).

### The LPP

Another promising component already tested in adults is the LPP, thought to index attention to emotional stimuli. More LPP positivity is typically found to unpleasant, high-arousal stimuli (Brown et al., 2013) interpreted as reflecting the “negativity bias” of rapid amygdala activation and processing to threatening stimuli (LeDoux, 1995). Importantly, “top-down” projections from the PFC can influence LPP responses; for example, Hajcak and Nieuwenhuis (2006) reported amplitude reduction when unpleasant stimuli were deliberately reappraised more positively. This has been aligned with reductions in amygdala activation and increases in PFC responses seen after mindfulness training (Goldin & Gross, 2010; Goldin, Ziv, Jazaieri, Hahn, & Gross, 2013). Indeed, Brown et al. (2013) found a strong inverse correlation between dispositional mindfulness and the LPP amplitude elicited by high-arousal unpleasant stimuli in a passive picture-viewing task. No research linking the LPP to mindfulness training has been conducted with adolescents, but it is known that LPP responses do not reach adult levels of modulation until late adolescence (Zhang et al., 2012). Peak emotional ratings of pictures and LPP amplitudes have been reported in young adolescents (Zhang et al., 2013), corresponding to the undeveloped nature of their ER abilities. It would be interesting to investigate whether mindfulness training can impact on the strength of emotional responses reflected in the LPP modulation in a young adolescent population.

### The CNV combined with the LPC

The last waveform of interest is the CNV, in conjunction with a late target stimulus-related ERP component, the LPC. During a negative-emotion “priming” task, more-negative CNV amplitudes to the warning stimulus followed by a reduced LPC could represent early preparation (Walter et al., 1964), followed by a reduction of secondary processing and rumination regarding that stimulus (Sitges et al., 2007). This ERP pattern could be used to map improvements in participants’ regulation through initial attention deployment and preparation (CNV), as has been seen in mindfulness research with depressed populations (Bostanov et al., 2012), followed by a withdrawal of appraisal and a reduction in rumination (LPC). Little research has looked into the potential of the LPC to signal adaptive ER, but tasks with affective stimuli manipulating an increase or reduction in rumination seem most suitable to test possible modulations of the LPC by mindfulness practice, with more-positive LPC amplitudes reflecting less adaptive (more ruminative) processing. Support for this approach has come from Rellecke et al. (2012), who observed that LPC amplitudes increased when participants willfully focused their attention on affective facial stimuli, indicating individuals’ voluntary control over higher-cognitive emotion processing. Interestingly, a dispositional study of Australian adolescents reported that higher levels of rumination exacerbate the relationship between self-reported life hassles and depression and anxiety, whereas higher mindfulness levels alleviated these same links (Marks, Sobanski, & Hine, 2010). Training young people in mindfulness techniques may therefore work to protect them against the damaging effects of rumination on well-being while dealing with everyday stressors. Potential future studies could examine whether such effects are detectable when measuring LPC and CNV ERP neural markers.

## Conclusions

We have argued that the neurodevelopmental perspective belongs to the frontiers of future research on mindfulness in adolescents, and the ERP methodology could prove particularly useful in this context. What is needed is an integrative approach in which (a) mindfulness programs are informed by neurodevelopmental trajectories and allow for adaptation in order to target developmentally salient challenges, such as disengaging from affectively salient stimuli or the lessening of peer pressure influences in adolescents; and (b) evaluations of mindfulness curricula focus on the aspects of neurocognitive function targeted by that particular mindfulness program. For example, an investigation of more-negative N2 amplitudes in response to interfering stimuli after mindfulness training, which would reflect more efficient disengagement that is linked to the development of an accepting attitude. Experiments utilizing ERPs can be particularly helpful here because of their functional sensitivity to shifts in attention control, the low cost of EEG equipment, and its portability, which enables more naturalistic experimental testing in schools, beyond the restrictive lab environment. To enable broader utilization of ERPs in developmental intervention studies on mindfulness, more basic research on reliable ERP markers is needed. This is particularly relevant to investigations of ER, since studies in both adults and children have been scarce. The initial research points to modulations of the LPP by emotion regulation strategies (Hajcak & Nieuwenhuis, 2006) and by trait mindfulness (Brown et al., 2013), but in the absence of longitudinal and developmental studies it is not clear how mindfulness as a unique ER strategy could modify the LPP component. It is also possible that with more mindfulness practice, the mindful ER strategy would lead to the modulation of ERP components (such as the P300) peaking earlier than the LPP. This would be due to shifts in more automatic responding to emotionally salient stimuli, as was suggested by adult fMRI research with beginners and long-term meditation practitioners (Taylor et al., 2011). Given the paucity of available research on ER with ERPs, measures of attention control and efficiency may be the most prudent outcomes to begin researching developmentally, since the ERP findings of mindfulness training in adults allow for more-confident predictions—that is, a more negative N2 (Moore et al., 2012) and less P300 positivity (Slagter et al., 2007).

In addition, researchers and mindfulness trainers need to work together when attuning mindfulness programs for developmentally and population- (e.g., gender-) specific features, such as adjustments to the frequency and length of home practices. Collaborations are needed in the evaluation process, to include converging assessments from self-reports and neurocognitive measures in combination with behavioral assessments from teachers and parents (where appropriate). And ideally, such research would inform education policy, focusing on investigating changes in variables that are high on school policy agendas, such as metacognition, attention, and well-being. An example would be to study changes in adolescent mind wandering after mindfulness training, which would investigate shifts in self-reported mind wandering together with modifications in target detection accuracy and P3b amplitudes reflecting attention efficiency. These findings could be linked to well-being assessments and behavioral reports of pupils’ self-regulation from teachers, as well as academic attainment records. Neurocognitive evidence would be key in this multidisciplinary approach, since only neurodevelopmental experimental paradigms can address focused questions regarding the endogenous trajectories of change in emotion regulation and attention within school-based mindfulness programs. In this way, neurocognitive research can provide pivotal evidence for education policy makers. A similar approach has been applied by the Neville lab to assess the neurocognitive basis of poor attention performance in children from lower socio-economic status (SES) backgrounds. These findings have guided focused interventions, which enhanced attention abilities in children from lower SES families (Stevens, Fanning, Coch, Sanders, & Neville, 2008).

To conclude, it is our hope that the considerations and research suggestions outlined in this article will inspire more researchers to adopt an integrative neurodevelopmental approach to the study of mindfulness, particularly using the ERP methodology. This will in turn boost our understanding of underlying mechanisms and help us fully harness the potential of mindfulness practice to improve well-being and support the neurocognitive development of adolescents.
